# Perspective on the paper: GDR MiDi. On dense granular flows

**DOI:** 10.1140/epje/s10189-026-00589-5

**Published:** 2026-06-29

**Authors:** Hans J. Herrmann, Stefan Luding

**Affiliations:** 1 Universidade Federal Do Ceara, Fortaleza, Brazil; 2 Department of Thermal and Fluid Engineering, University of Twente, Enschede, The Netherlands

## Abstract

The study ***"On dense granular flows"*** by GDR MiDi, published in 2004 in The European Physical Journal E 14, 341–365, stands as a seminal collective work that significantly advanced the understanding of dense granular materials. Remarkably, the names of the contributing authors are not listed; only the name of the consortium is given—a brave statement for joint efforts like this!. The GDR Midi (Groupement de Recherche sur les Milieux Divisés) was a CNRS-led consortium of French laboratories. Its mission was to bring together diverse scientific communities—including solid mechanics, fluid mechanics, physics, and geophysics—around the study of granular media, in order to promote collaboration through frequent, informal meetings held typically four times per year (see group photograph of a meeting in Carry Le Rouet in 2005). This approach enabled the identification of key scientific challenges as the need to compare and consolidate data across different configurations, between experiments and simulations, and across different research groups, ultimately inspiring the work that led to the collective GDR Midi paper.

**Graphical abstract:**

**Figure:**
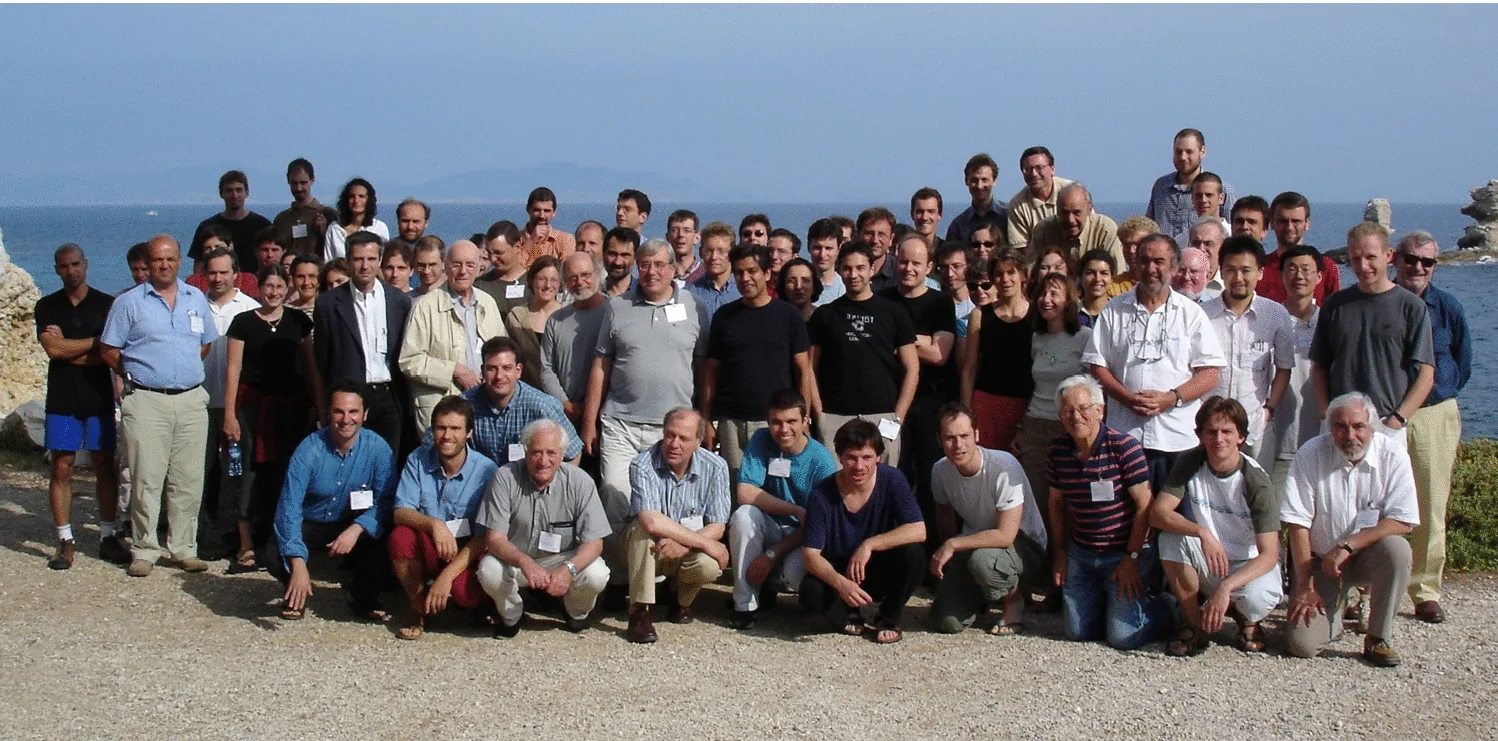
Group photograph of the GdR Midi meeting 2005 in Carry Le Rouet. This perspective will explore its initial importance, its enduring relevance as reflected by more than 2500 citations [google scholar, Jan. 2026], and the future directions it continues to inspire.

## Retrospective: a landmark in granular physics (2004)

At the turn to the twenty-first century, the field of granular physics was experiencing a surge of interest, yet fundamental gaps remained related to the understanding of dense granular flows. While two extreme regimes were relatively well-characterized—the quasi-static regime, often described by soil plasticity models (e.g., Mohr–Coulomb, Drucker–Prager) [1, 2], and the dilute gaseous regime, based on the dissipative analogy to kinetic theory for rapid gas flows [3, 4]—the intermediate “dense flow” or “liquid” regime presented a formidable challenge. This regime, where grain inertia becomes significant, but a pervasive contact network still exists, lacked a unifying constitutive description. In fact, the idea that a network of persisting contacts fully characterizes dense granular flows is too simple. As the authors themselves stated, *“Up to now no constitutive equations are available in this 'liquid' regime and no unified framework allows to describe the whole dynamics from quasi-static to gaseous regime”* [5]. Compounding this issue was the fragmentation of research, with numerous studies employing diverse experimental setups and numerical methods, often focusing on one or the other special case only, making systematic comparisons and the extraction of universal principles notoriously difficult.

It was into this landscape that the GDR MiDi paper emerged as a game-changing contribution. Far from being a mere collection of isolated results, it represented a monumental “collective work” by the Groupement de Recherche des Milieux Divisés (GDR MiDi) [5], a French research consortium with focus on particulate media. Their collaborative approach, pooling data from various laboratories, experiments, and numerical simulations across France, was unprecedented in its scope, width, and depth. The stated goal was explicit: “*… to achieve a coherent presentation of the relevant quantities … in six different geometries”* [5]. Such a systematic, comparative study was precisely what the field needed to move beyond fragmented observations toward a cohesive understanding.

The authors meticulously analyzed steady, uniform granular flows in six distinct geometries: plane shear, annular shear, vertical-chute flows, inclined planes, heap flows, and rotating drums, see Sect. 2 in Ref. [5]. This breadth allowed the researchers to identify *“robust features”* that transcended specific experimental setups. Among its most crucial findings was the identification of the inertial number, $I=\dot{\gamma }d\sqrt{P/\rho }$, as a key dimensionless parameter governing the behavior of dense granular flows, see Eq. (1), Sect. 3.1., in Ref. [5]. Here, $\dot{\gamma }$ is the mean shear rate, d is the particle diameter, P is the confining pressure, and ρ is the material density. For typical granular materials (where the particles are rigid, the restitution coefficient e is not too close to unity and interparticle friction ${\mu}_{p}$ is not too small), many macroscopic flow properties—including volume fraction profiles, velocity profiles, and effective friction—were found to be primarily controlled by I, see Sects. 3.2 and 3.3 in [5]. This insight provided the much-needed bridge between microscopic particle properties and macroscopic flow characteristics, offering a pathway toward a continuum description, echoing early attempts like Bagnold's [6] but within a more comprehensive framework for dense flows. Note that the inertial number is very similar to the Savage number: $Sa={I}^{2}$ introduced a few years earlier [7].

The concept of effective friction, ${\mu}_{\mathrm{e}\mathrm{f}\mathrm{f}}$, was thoroughly explored, revealing its complex dependence on I, Figs. 2e, 3b, 5e, 8 in [5]. In the quasi-static limit (small I→0), ${\mu}_{\mathrm{e}\mathrm{f}\mathrm{f}}$ is rate-independent, akin to Mohr–Coulomb bulk friction. As I increases into the dense inertial regime, ${\mu}_{\mathrm{e}\mathrm{f}\mathrm{f}}$ increases linearly, indicating a rate-dependent behavior. At even larger I, effective friction saturates to the dilute collisional regime, sensitive to the restitution coefficient e, Sects. 3.3, 8.2 in [5]. This behavior was summarized into the empirical $\mu \left(I\right)$ relationship; derived from their extensive comparative analysis [5], it became the cornerstone for the development of modern continuum granular rheology [8, 9]. One missing ingredient, a relation for the volume fraction $\phi \left(I\right)$ was only touched in Fig. 2(f) in [5], but was much studied in the following years to establish the more complete the $\mu \left(I\right)-\phi \left(I\right)-$ rheology adding not only compressibility effects but also viscosity to the picture via the viscous inertial number ${I}_{v}$ [8].

Kinematically, a comprehensive catalog of observed velocity profiles across various geometries was provided, Sects. 3.2.1, 4.3.1, 5.2.1, 6.3.1, 7.3.1 in [5]. The prevalence of shear localization was highlighted, where deformation is concentrated in narrow bands, particularly in confined flows (e.g., annular shear, vertical-chute flows), see Sects. 4.3.1, 5.2.1, 8.3 in [5]. This phenomenon, previously observed in various forms, e.g., Savage, 1984 [10], was systematically documented also in other geometries, such as inclined planes, displaying Bagnold-like profiles or exponential tails, Sects. 6.3.1, 7.3.1 in [5]. Having these diverse kinematic features reported together, across different configurations, was important for future model development and validation. Aligned with this hydrodynamic approach significant progress has since been made also in continuum modeling of segregation [11].

Furthermore, the GDR MiDi study shed light on the hysteretic nature of granular flows, particularly concerning flow initiation and cessation, Sects. 4.2, 6.2, 7.2, 8 in [5]. Distinct critical angles (or torques) were identified for starting versus stopping flow, showcasing the complex history dependence inherent to granular matter, a topic explored in earlier works like those on avalanche dynamics [12]. The authors also further clarified the role of earlier discussed microscopic parameters [13]. While interparticle friction, ${\mu}_{p}$, and restitution coefficient e influence thresholds and transitions between regimes, the study concluded that these microscopic timescales had *“no influence on the macroscopic flow properties”* when sufficiently separated from the bulk flow timescales, Sect. 8.1.1 in [5]. This simplification was an essential ingredient for various macroscopic theories.

Perhaps most presciently, the GDR MiDi paper offered an early glimpse into the limitations of purely local rheologies and hinted at the necessity to include non-local effects. While the μ(I) concept successfully unified many average properties, the authors noted discrepancies when trying to predict detailed velocity profiles, especially in flows like heap flows where a *"coherence length"* ${\ell}(y)$ was observed to vary across layers, Sect. 8.4.3 in [5]. The explicit statement *"… will force us to relax the local-rheology hypothesis … ,"* Sect. 8.4.1 in [5], was foreshadowing, a major area of research of subsequent decades, see e.g., [14].

In summary, the GDR MiDi paper was indispensable when published because it provided critical knowledge on dense granular flows through an unprecedented collaborative effort. It unified disparate observations, identified the crucial dimensionless inertial number, characterized effective friction and kinematic profiles across diverse setups, and, most importantly, provided a foundational empirical basis for continuum rheology, while simultaneously flagging the inherent non-local complexities that would define the field's future trajectory. It served as both a comprehensive foundation and a powerful catalyst for subsequent research.

## State of the field: enduring legacy and evolving relevance

Twenty years after its publication, the GDR MiDi paper continues to resonate deeply within the field of granular physics, its contributions serving as both foundation and persistent point of reference. The granular field itself has matured considerably, advanced numerical methods, and diverse applications. This evolution, in many ways, was directly propelled by the insights and challenges first elucidated in the GDR MiDi work.

The most enduring legacy of the paper lies in its pivotal role in establishing the μ(I) rheology (or inertial rheology) as the de facto standard for describing dense, dry, cohesionless granular flows of rigid spheres. The systematic demonstration that the inertial number I provided a universal dimensionless parameter for many macroscopic flow properties was a groundbreaking realization, Sect. 8.4 in [5]. This empirical relationship, where the effective friction ${\mu}_{\mathrm{e}\mathrm{f}\mathrm{f}}$ is a function of I, offered the first widely applicable continuum constitutive law for the previously elusive dense flow regime. Today, the μ(I) rheology and its various extensions and derivations are routinely taught in granular physics courses, implemented in computational fluid dynamics (CFD) codes [15], and applied across industrial and geophysical contexts, from silo discharge to landslide modeling [16]. The GDR MiDi paper's meticulous comparative study across multiple geometries provided the robust empirical validation that cemented the $\mu \left(I\right)$ model's credibility and widespread adoption. It showed that despite apparent differences in flow behavior, a common underlying physical mechanism, characterized by I, could unite them.

Beyond the μ(I) law itself, the paper's meticulous collection and synthesis of experimental and numerical data from diverse flow configurations have remained an invaluable benchmark dataset, see Tables 1–4 in [5]. New theoretical models, novel numerical simulations (e.g., using the discrete element method, DEM) [17, 18], and advanced experimental techniques (e.g., X-ray tomography, particle image velocimetry, MRI) continue to refer to the *"robust features"* and specific data presented in the GDR MiDi work for reference and validation. The detailed velocity profiles, volume fraction profiles, and effective friction curves, which were painstakingly compiled, offer a rigorous test bed for any aspiring new theory of granular flow. Without such a comprehensive, comparative analysis [5], the fragmentation of results would have made progress far more arduous.

The paper's foresight regarding non-local effects is particularly striking in hindsight. While the μ(I) rheology successfully captured average flow properties, the GDR MiDi authors explicitly noted its limitations in predicting detailed velocity profiles, particularly in situations where the *"coherence length"* varied across the flow, leading to deviations from local predictions, e.g., in heap flows or near boundaries, Sect. 8.4.3 in [5]. This observation, coupled with the explicit call to *"relax the local rheology hypothesis"* [5], was a foundational step in identifying non-locality as a critical, unsolved problem. Today, non-local granular rheologies constitute one of the most active and challenging areas of granular physics. Models such as non-local fluidity models, see e.g. Refs. [19, 20], and others, aim to incorporate length scales that go beyond the particle diameter, directly addressing the types of discrepancies first highlighted by GDR MiDi. The *"coherence length"* concept introduced in the paper can be seen as a direct precursor to the *"healing length"* or *"cooperativity length"* scales in modern non-local theories, which account for the spatial correlations of particle rearrangements, e.g., Nicolas et al., 2018 [21]. Recently it was claimed by Berzi [22] that inertial rheology and non-local models are special cases of kinetic theory.

The paper's systematic documentation of shear localization across various confined geometries also retains its relevance, Sects. 4.3.1, 5.2.1, 8.3 in [5]. The understanding of why shear bands form, their thickness, and their interaction with boundaries is central to many applications and fundamental questions. The GDR MiDi data provided empirical evidence that any comprehensive granular rheology must be able to predict and explain these localized shear zones, a feature that local μ(I) models often struggle with without additional considerations.

Furthermore, the collaborative spirit exemplified by the GDR MiDi work has continued to influence the field. The necessity of cross-institutional and multi-method approaches (combining experiments, simulations, and theory) for tackling complex granular phenomena is now widely accepted. Also, other disciplines contributed as for example the mathematical studies on well- and ill-posedness of the μ(I) rheology—and how to deal with it [23].

The GDR MiDi author's emphasis on standardizing *"relevant quantities to be measured,"* see Introduction, Sect. 9 in [5], also contributed to fostering a more unified and productive research community, as exemplified by the COST network OnDEM [https://www.cost.eu/actions/CA22132/].

In essence, the GDR MiDi paper, published in 2004, was not just a summary of findings; it was a blueprint for future research. It provided essential tools (like the dimensionless inertial number I) for a macroscopic description, a wealth of data for validation, and a clear articulation of some of the field's grand challenges (e.g., non-locality, shear localization). Its insights remain deeply embedded in the theoretical frameworks, computational models, and experimental methodologies employed in granular physics today, underscoring its profound and enduring relevance.

Nevertheless, there are recent research developments that only now fill some of the gaps that naturally are left by an original work as by GDR MiDi [5]. For cohesive powders, various additional questions need to be addressed, as discussed in a most recent review by Pouliquen, 2025 [24]. In addition to cohesion, various other mechanisms (like softness, fluid–viscosity, free surface, or creep effects) can be relevant and thus have to be embedded in an improved rheology by additive or multiplicative correction terms [25, 26]. Progress toward viscous-cohesive systems was achieved by a unified inertial number ${I}_{m}$ resulting in the $\mu \left({I}_{m}\right)-\phi ({I}_{m})$ rheology [25]. Quantifying additional effects by other dimensionless numbers, as e.g., compressibility ${P}^{*}$, provides generalized $\mu \left(I,{P}^{*}, \dots \right)-\phi (I,{P}^{*},\dots )$ relations, so far for dry flows [26], where the correction terms vanish if the mechanism is not relevant.

## Future directions: navigating the granular frontier

The legacy of the GDR MiDi paper provides a robust foundation, yet also illuminates numerous avenues for future exploration in granular physics. Building on the μ(I) rheology and the recognized limitations of purely local descriptions, the field is ready for advancements in several key areas.

One of the most critical future directions is the development and robust validation of universal non-local granular rheologies. While significant progress has been made [14, 20], a universally accepted and computationally tractable non-local constitutive law, capable of accurately predicting shear band formation, flow initiation, and boundary effects across diverse geometries and flow conditions, remains elusive, but is the focus in recent research, see e.g., [27]. Future research will need to move beyond scalar or simple gradient theories to more complex formulations, possibly incorporating additional internal variables that reflect microstructural changes (e.g., fabric anisotropy, contact network evolution, rotations, shape, etc.) or concepts akin to granular temperature or fluidity fields. Experimentalists will need to devise novel measurements of these variables, while simulators will require more sophisticated DEM techniques to extract and coarse grain/homogenize micro-scale information into continuum-level descriptions. The *"coherence length"* first discussed in GDR MiDi, Sect. 8.4.3 in [5], will continue to be a central concept in this quest.

Another significant frontier involves moving beyond steady, uniform flows to understand dynamic and transient granular phenomena. The GDR MiDi paper primarily focused on steady states, but recognized the importance of transient behaviors, Sects. 4.2, 6.2, 7.2 in [5]. Future research should study the coexistence and the dynamics of transitions between the quasi-static, dense inertial, and dilute collisional regimes, as well as the rapid propagation of disturbances and instabilities within granular flows. This will require developing time-dependent, possibly non-local rheologies—as well as advanced numerical methods that can handle large deformations and rapid changes in flow states.

The influence of particle shapes and interparticle forces beyond simple dry, cohesionless, and nearly monodisperse spheres will become increasingly important, see e.g. Refs. [28, 29]. The GDR MiDi authors discussed these parameters, Sect. 8.1.1 in [5], but noted their limited effect for the specific conditions studied, implying future work for other conditions with additional mechanisms [25, 26]. Future work will explore how these complexities modify the $\mu \left(I\right)$ relationship and non-local effects, necessitating the development of multi-scale rheologies [30]. This has direct implications for industrial processes (e.g., pharmaceutical powders, food processing) and geophysical hazards (e.g., mudslides).

A rapidly expanding area is the study of fluid–granular interactions. While GDR MiDi focused on dry granular flows, many practical scenarios involve granular materials immersed in or interacting with fluids (e.g., fluidized beds, suspensions, debris flows, submarine landslides). In fact, the importance of considering pressure-imposed rheologies has proven both necessary and fruitful in suspension rheology [8, 31]. Developing unified rheologies for such two-phase flows, which account for drag, added mass, and pore pressure effects, is a grand challenge [32]. This requires bridging granular physics with fluid mechanics, often through coupled CFD-DEM [33] or continuum mixture models.

Computational granular mechanics will continue to play a pivotal role. The GDR MiDi authors extensively used numerical simulations, Tables 1–4 in [5], setting a precedent. The increasing power of DEM simulations combined with continuum models [34] will enable studies of larger systems and more complex particle interactions, providing deeper insights into the micro-macro link. Also, machine learning and artificial intelligence are emerging as powerful tools for discovering novel constitutive laws from vast datasets (both simulated and experimental), optimizing parameters for existing rheologies, and predicting complex flow behaviors without explicit constitutive models, see e.g. Ref. [35]. Data-driven approaches could revolutionize how granular rheologies are developed and implemented in the future.

A deeper fundamental theoretical understanding of the micro–macro link remains a core challenge. While empirical rheologies like μ(I) are effective, a robust statistical mechanics or field theory of granular matter, capable of deriving macroscopic laws from microscopic interactions and capturing non-local effects, is still an active area of theoretical physics, see e.g. Refs. [36, 37]. This involves understanding how local particle rearrangements and contact network dynamics give rise to continuum stresses and strains, as highlighted by GDR MiDi's call to understand *"the origin of non-locality and its relationship to the rheology,"* Sect. 9 in [5].

Finally, the translation of fundamental understanding into robust and predictive engineering models is crucial for wider impact. This means developing computationally efficient and stable numerical solvers for advanced granular rheologies, enabling their use in real-world industrial design (e.g., silo optimization, conveying systems, mixers) and geophysical hazard mitigation (e.g., predicting landslide initiation and runout, dam break flows, etc.). Continued efforts in standardization of experimental protocols and data sharing will further accelerate progress, mirroring the collaborative spirit that made the original GDR MiDi paper so impactful, Sect. 9 in [5]. In the same spirit, recent collaborative frameworks started to pave the way for future particle simulation models—in particular the open source complementing commercial software developments.

In conclusion, the GDR MiDi paper has set the stage for modern granular physics. Its legacy demands that future research not only refines existing models but also pushes the boundaries into more complex, descriptions, searching for more universal, possibly non-local constitutive laws grounded in a deeper understanding of the micro-mechanics and physics of granular matter and in particular the micro–macro connection. The granular frontier remains vibrant and expansive, continually challenging researchers to unify their efforts.
