# Correction: Perspective on the paper: GDR MiDi. On dense granular flows

**DOI:** 10.1140/epje/s10189-026-00624-5

**Published:** 2026-08-21

**Authors:** Hans J. Herrmann, Stefan Luding

**Affiliations:** 1 Universidade Federal Do Ceara, Fortaleza, Brazil; 2 Department of Thermal and Fluid Engineering, University of Twente, Enschede, The Netherlands

**Correction to: The European Physical Journal E (2026) 49:55**

**https://doi.org/10.1140/epje/s10189-026-00589-5**

During the preparation of this perspective, the authors performed a literature review and accidentally included some incorrect references without sufficient checking the details.

This resulted in 16 references of the original article being incorrect.

The authors apologize for the oversight and have corrected the article, which now reports the correct reference list.

The original article has been corrected.

